# Oxidation Products of Tryptophan and Proline in Adipokinetic Hormones—Artifacts or Post-Translational Modifications?

**DOI:** 10.3390/life13122315

**Published:** 2023-12-10

**Authors:** Simone König, Heather G. Marco, Gerd Gäde

**Affiliations:** 1 IZKF Core Unit Proteomics, Interdisciplinary Center for Clinical Research, University of Münster, Röntgenstr. 21, 48149 Münster, Germany; 2 Department of Biological Sciences, University of Cape Town, Private Bag, Rondebosch, Cape Town 7700, South Africa; heather.marco@uct.ac.za (H.G.M.); gerd.gade@uct.ac.za (G.G.)

**Keywords:** mass spectrometry, peptide fragmentation, hydroxyproline, kynurenine

## Abstract

Background: Adipokinetic hormones (AKHs) regulate important physiological processes in insects. AKHs are short peptides with blocked termini and Trp in position 8. Often, proline occupies position 6. Few post-translational modifications have been found, including hydroxyproline ([Hyp^6^]) and kynurenine. Our recent data suggest that the Hyp- and Kyn-containing AKHs occur more often than originally thought and we here investigate if they are natural or artifactual. Methods: From crude extracts of the corpora cardiaca (CC) of various insect species, AKHs were analyzed using liquid chromatography coupled to high-resolution mass spectrometry (LC-MS). Synthetic [Hyp^6^]-AKHs were tested in an in vivo metabolic assay. Freshly dissected *Periplaneta americana* and *Blaberus atropos* CCs (with precautions taken against oxidation) were analyzed. *B. atropos* CC were placed into a depolarizing saline and the released AKHs were measured. Results: Hyp was detected in several decapeptides from cockroaches. The modified form accompanied the AKH at concentrations below 7%. The [Hyp^6^]-AKHs of *B. atropos* were present in fresh CC preparations and were shown to be releasable from the CC ex vivo. Synthetic [Hyp^6^]-containing peptides tested positively in a hypertrehalosemic bioassay. Hydroxyprolination was also detected for Manto-CC from the termite *Kalotermes flavicollis* and for Tetsu-AKH of the grasshopper, *Tetrix subulata*. Oxidized Trp-containing forms of Nicve-AKH were found in species of the burying beetle genus *Nicrophorus*. Conclusions: Trp oxidation is known to occur easily during sample handling and is likely the reason for the present findings. For hydroxyprolination, however, the experimental evidence suggests endogenous processes.

## 1. Introduction

**Figure 1:**
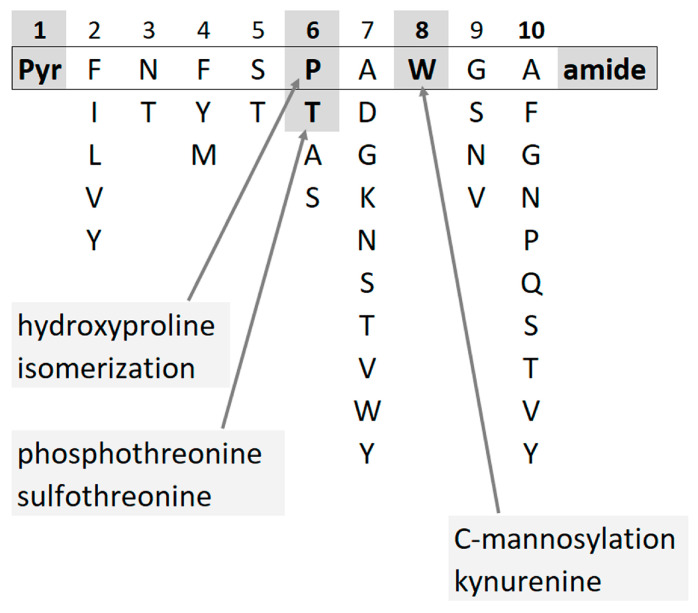
AKHs have conserved structural features such as blocked termini and specific amino acid residues in each position. Few modifications have been described so far.

In insects, neuropeptides regulate processes such as homeostasis, development, reproduction, and behavior [1,2]. One of the peptide hormone families, the so-called adipokinetic hormone (AKH)/red pigment-concentrating hormone (RPCH) family, is involved in energy metabolism making stored metabolites available for use in the hemolymph of insects; more than 100 members are known with conserved structural features [3,4]. Mature AKHs are eight to ten amino acids long, with blocked N- (pyroglutamate, pQ) and C- (carboxyamide) termini and specific amino acid residues in each position. Tryptophan is always localized at position 8, and in more than half of all AKHs, a proline residue occupies position 6. A few modifications of AKH peptides have been described, viz. phosphothreonine [5], sulfothreonine [6], C-mannosylation at the tryptophan residue [7,8], proline isomerization (putative) [9], as well as oxidative changes on the Trp and Pro residues, i.e., kynurenine (Kyn) [10] and hydroxyproline (Hyp) [11,12] (Figure 1).

The finding of a Hyp form of an AKH in diverse insect orders such as Hemiptera and Diptera [11,12] formed the basis of a closer inspection for this peptide modification in subsequent corpora cardiaca (CC) samples. In many cockroach species (order: Blattodea), Hyp-modifications were commonly detected but not verified for most of the [Pro^6^]-containing AKHs [13].

Trp oxidation of AKHs, on the other hand, is under-reported, but the phenomenon of Trp oxidation in other proteins and peptides may be the result of in vivo processes—some of which may be detrimental to health, some may be neutral, and some may act as a signal (for review, see [14,15]). However, Trp oxidation can also be an artifact from sample handling (see Section 1.2 below). It is, therefore, imperative to establish whether an observed modification to a peptide/protein is a natural product or a result of an artificially induced process. Critically, peptide oxidation may occur as an artifact of the ionization process during the electrospray ionization high-resolution mass spectrometry (MS) experiment, and is then detected at the same retention time as the unmodified species [16,17,18]. Naturally, modified peptides (meaning, biologically endogenous as opposed to those with an artificial origin from the MS procedure) will appear at slightly different retention times from those of the unmodified (“parent”) peptide species in liquid chromatography (LC), which separates the analyte peptides before MS.

The biochemical factors behind the oxidative processes that give rise to Hyp and Kyn are supplied below for a better understanding.

### 1.1. Hydroxyproline

Hyp is present in animals mainly as *trans*-4-hydroxy-l-proline and its minor analog *trans*-3-hydroxy-l-proline in a ratio of ~100:1 [19]. Prolyl hydroxylation is the most common post-translational modification in humans and is known for the stabilizing function of Hyp in the collagen triple helix [20]. The reaction is irreversible and catalyzed by prolyl 4-hydroxylase (P4H), an enzyme that has been described not only in animals but also in plants and microbes (for a review on P4H, see [20]). Hyp is found to a lesser extent in non-collagen proteins; it is of structural and physiological significance, e.g., for scavenging reactive oxygen species (for more information on Hyp structure and function, see [19,21]).

The minimum substrate required for enzyme recognition is described as XPG with the PPG sequence showing the highest hydroxylation rates in some studies [20,22]. In [Ala^2^]-bradykinin, however, relative hydroxylation was almost three times better for APG than for PPG, and some dependency in oxidation efficiency from N-terminal modifications of the short peptide was observed [23]. In mammalian and frog hypothalamus, [Hyp^9^]-luteinizing hormone-releasing hormone was detected with the amino acid residue triplet R-Hyp-G [24]. In HeLa S3 cells, besides PPG, also SPG, SPA, TPN, SPE, DPV, and APS sites were hydroxylated [25]. It thus seems that Gly in position 3 of the triplet is not a universal requirement. This was also seen in the case of toxins of the marine gastropod mollusk genus *Conus* that are reported to contain T-Hyp-Hyp-K, P-Hyp-K, T-Hyp-P, T-Hyp-Hyp-R, P-Hyp-R, K-Hyp-Q, R-Hyp-T, and D-Hyp-R [26,27,28].

Little is known about hydroxyprolination in insects. The first report of a hydroxyprolinated AKH ([Hyp^6^]-Panbo-RPCH, code-named Nezvi-AKH) was in 2011 from the CC of the green stink bug *Nezara viridula*, with Panbo-RPCH (pQLNFSPGW amide) as the “parent peptide” being modified [11]. Eleven years later, this modification was shown for an AKH in the horse fly *Haematopota pluvialis*: the hydroxyprolinated Tabat-AKH (pQLTFTP GW amide) was code-named Haepl-AKH [12]). Extensive experiments with Nezvi-AKH, including extraction in an oxygen-free atmosphere, were performed to exclude artifactual Pro oxidation [11]; such checks were not carried out with Haepl-AKH.

There is, however, not much evidence in the literature that Hyp is easily formed at ambient conditions—in contrast to spontaneous methionine oxidation during sample handling, which has been abundantly proven for peptides and proteins [29]. Apparently, the presence at least of oxidizing agents is required to modify Pro; this residue in human apolipoprotein B-100 was highly reactive toward oxygen radicals in vitro for two different oxidation systems in the presence of low iron concentrations [30]. Radical attack (copper and H_2_O_2_) also modified Pro residues in a cell lysate of primary cultures of chick embryo myotubes [31], but in these experiments, the involvement of enzymes could not be excluded.

Using the search term “prolyl hydroxylase”, we extracted 750 entries for this enzyme in insects from the Uniprot protein database. Except for one result (Q9I7H9, *Drosophila melanogaster*), they were all unreviewed entries, which were assigned to the enzyme class by sequence similarity to known proteins with little experimental backing. *D. melanogaster sudestada1* (*sud1*), however, was identified as a gene that is needed for normal growth in the fly [32]. *Sud1* encodes a prolyl-hydroxylase that catalyzes post-translational hydroxylation of a conserved residue in the small ribosomal subunit protein RPS23; knockdown of *Sud1* results in growth impairment and reduced RPS23 hydroxylation, which is associated with activation of the unfolded protein response, induction of apoptosis, and increased autophagy [32].

### 1.2. Tryptophan Oxidation

**Figure 2:**
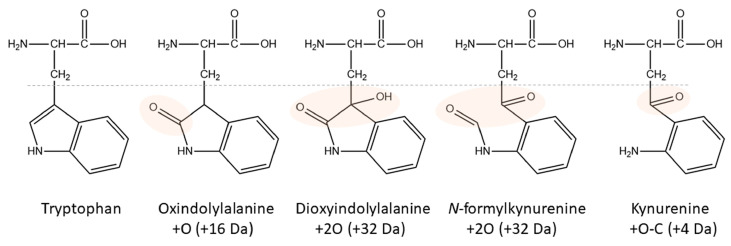
Structures of Trp and its major oxidation products. Modified areas are highlighted. The corresponding nominal mass increase is given in brackets.

Ten years ago, a Kyn-containing variant of an AKH formerly identified as a hypertrehalosemic hormone in the Indian stick insect *Carausius morosus* (Carmo-HrTH [33]) was detected as pQLTFTPN-Kyn-GT amide in the Vietnamese stick insect, *Baculum extradentatum* [10]. At the time, it was thought to be a post-translational modification, but mounting evidence and the present study suggest that it is more likely to be a handling artifact. The indole ring in Trp is highly reactive (primarily the pyrrole ring [34]) and this amino acid residue is susceptible to oxidation and degradation into multiple products during sample preparation [35]. Factors like reactive oxygen species (singlet oxygen, hydrogen peroxide, hydroxyl radicals), light and photosensitizers, metals, and heat may contribute to these processes (for an introductory review, see [35]). The stability of Trp-containing products is a well-known problem in the food (e.g., milk proteins [36,37]) and textile industries [38] as well as for pharmacological preparations (e.g., monoclonal antibodies [39]). Kyn is a common oxidation product in addition to singly, doubly, and triply oxidated forms (Figure 2), but even more Trp modifications have been described [35,40].

In an earlier investigation, difficulties arose when studying the actions of α-melanocyte stimulating hormone (αMSH) and αMSH(1-12) (_Ac_SYSMEHFRWGKPV amide) in cell culture, because the Trp residues of the hormone were oxidized to at least five different products [41]. Peptide oxidation was slowed by the addition of tris-(2-chloroethyl) phosphate, a very effective reducing agent, but its use was limited by its cell toxicity at higher concentrations. Interestingly, no special chemicals such as hydrogen peroxide or irradiation (as found in other studies: photooxidation [40,42,43], ozone [44,45], and gel electrophoresis procedures [46]) were necessary to modify the Trp residue in experimentation with αMSH and αMSH(1-12); it sufficed to keep the peptides at room temperature for some time [41]. Thereby, Trp oxidation took longer than Met oxidation and was not as dominant at higher peptide concentrations.

Trp oxidation has also been described in in vivo processes. For instance, exposure of *Arabidopsis thaliana* plants to light stress resulted in an increased level of oxidized Trp in proteins of the photosystem II reaction center and the oxygen-evolving complex [47]. Increased levels of oxidative stress are also believed to play a key role in the development of age-related diseases through the oxidation of amino acid residues, and this was substantiated for Trp in α-skeletal actin and troponin I using a rat model of acute oxidative stress induced by X-ray irradiation [48]. Both of these studies, however, used gel electrophoresis for protein separation before analysis, which was shown to oxidize Trp [46]. In other work, modified Trp residues were detected with LC-MS in cyclic microcystin peptides prepared from cyanobacterium *Microcystis* sp. CAWBG11 using precautions to avoid artificial oxidation [49].

Trp oxidation does not always require an enzyme to catalyze it, although such processes are also known from the Kyn pathway. Tryptophan 2,3-dioxygenase (TDO) and indoleamine 2,3-dioxygenase are members of a small family of heme enzymes that catalyze the aerobic metabolism of _L_-Trp to N-formylkynurenine in both eukaryotes and prokaryotes [50]. While these two enzymes have only been described to act on Trp or some of its small molecule derivatives, a peptide-tryptophan 2,3-dioxygenase, also called pyrrolooxygenase (PO), has been reported from plant and animal sources, which forms peptide formylkynurenine [51,52]. For TDO in insects, several entries were found in Uniprot, such as P20351 for *D. melanogaster* and Q17P71 for *Aedes aegypti*, but none for PO.

Thus, the aims of the current study were: (1) to validate the [Hyp^6^]-modified AKH sequences recently observed in cockroaches [13] with the use of synthetic peptides and LC-MS, (2) to ascertain if these peptides retain biological activity in a metabolic assay, and (3) to elucidate whether the prevalence of the modification is an artifact arising from peptide handling, or whether the oxidated form is already present in the CC itself (and, thus, releasable ex vivo). Furthermore, (4) the investigations to detect other oxidized AKHs, specifically Trp oxidation, were extended to beetles (Order: Coleoptera) through the availability of CC extracts from burying beetle species of the genus *Nicrophorus*.

## 2. Results and Discussion

### 2.1. Hydroxyproline

#### 2.1.1. MS Analysis

In recent investigations, AKH candidates were identified by target-MS (fragmentation of pre-selected peptide ions) for eligible known peptide masses from related insect species or as calculated from genomic/transcriptomic sequence data [12,13]. The targeted MS approach also makes use of marker fragment ions discovered for Pro-containing AKHs, i.e., three dominant fragment ions in collision-induced dissociation (CID) spectra that arise from the special way in which an AKH with [Pro^6^] breaks during CID as a consequence of the rigid ring structure of Pro [53]. In this way, Hyp in the octapeptide Haepl-AKH of the horse fly *H. pluvialis* was demonstrated and validated with a corresponding synthetic peptide [12], and several [Hyp^6^]-modified AKHs were sequenced from a variety of cockroach species, including the validation of [Hyp^6^]-Tenmo-HrTH in *Ergaula capucina* [13]. In these instances, the trio of marker ions originate from cleavage from both ends of the molecule and some still contain the Pro residue so that the nominal mass shift of 16 mass units proved the position of the modification [12,13,53]. With this surprising trove of hydroxyprolinated AKHs and the knowledge that the modified AKH ion signals are not as prominent as those of the parent AKH, we reanalyzed recent CC samples on LC-MS to detect and validate [Hyp^6^]-AKHs.

**Figure 3:**
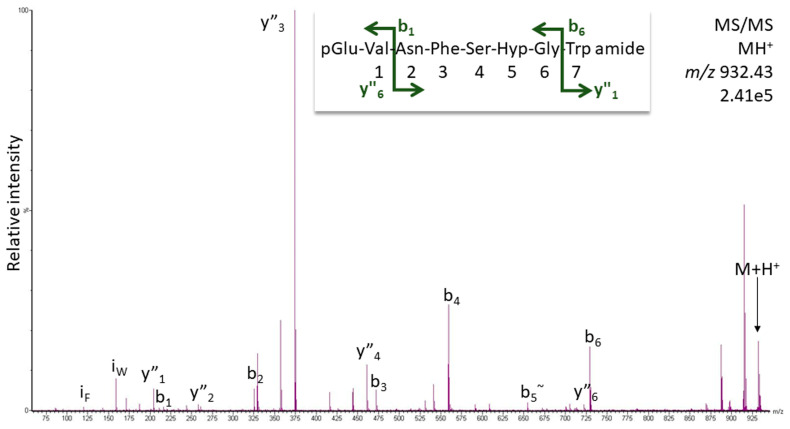
MS/MS analysis for [Hyp^6^]-Manto-CC in *K. flavicollis* using the singly charged peptide ion (*m*/*z* 932.43). Peaks were labeled according to the b- and y-ion series as calculated in Supplementary Figure S1. For original spectrum and validation with the synthetic compound, see Supplementary Figure S2.

From the CC extract of the termite *Kalotermes flavicollis*, hydroxyprolination was found for the octapeptide Manto-CC (Figure 3). The fragment ion pattern of the modified peptide ([Hyp^6^]-Manto-CC) paralleled that of Manto-CC [12] with the expected shift of nominal 16 Da for Hyp-containing ions. For the [Hyp^6^]-Manto-CC fragmentation seen in Figure 3, these are y″_3_, y″_4_, and b_6_ (*m*/*z* 358.19 to 374.18, 445.22 to 461.21, and 713.326 to 729.32; compare expected fragment ion masses in Supplementary Figure S1). Other dominant ions such as b_4_ do not change in mass upon modification (*m*/*z* 559.25) and, thus, only contribute to the identification of the peptide.

The Hyp form of the AKHs eluted about 1 min earlier in LC than the respective AKH itself [12]. The sequence identity of [Hyp^6^]-Manto-CC in *K. flavicollis* was validated using the synthetic compound and the same LC-MS method; both the CID spectra and retention times agreed (Supplementary Figure S2).

**Figure 4:**
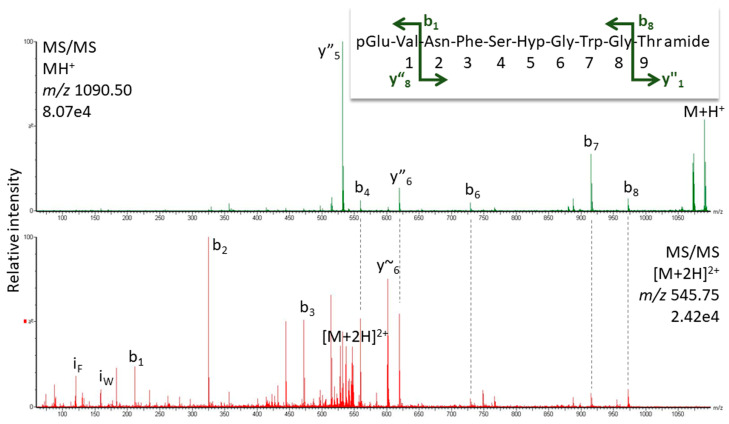
MS/MS analysis for the singly and doubly charged peptide ions (*m*/*z* 1090.50 and 545.75) of a peptide assigned to [Hyp^6^]-Bladi-HrTH in *Xestoblatta cavicola*. Peaks were labeled according to the b- and y-ion series as calculated in Supplementary Figure S3. For original spectra, see Supplementary Figure S4.

**Figure 5:**
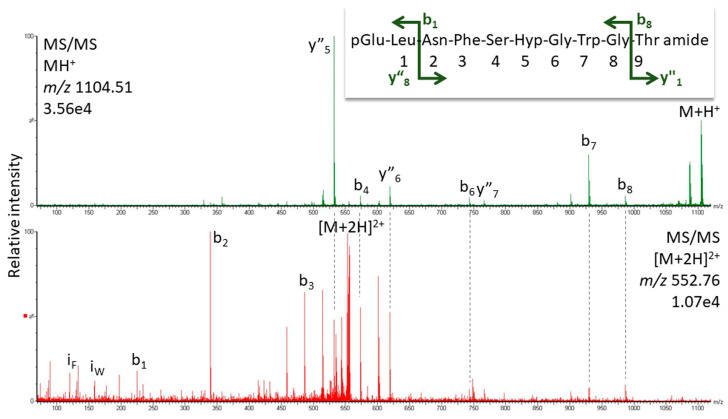
MS/MS analysis for the singly and doubly charged peptide ions (*m*/*z* 1104.51 and 552.76) of a peptide assigned to [Hyp^6^]-Panni-HrTH in *Panchlora nivea*. Peaks were labeled according to the b- and y-ion series as calculated in Supplementary Figure S6. For original spectra, see Supplementary Figure S7.

**Figure 6:**
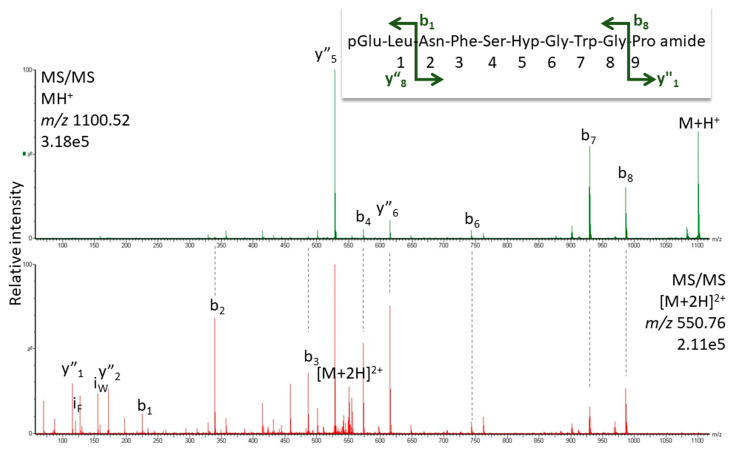
MS/MS analysis for the singly and doubly charged peptide ions (*m*/*z* 1100.52 and 550.76) of a peptide assigned to [Hyp^6^]-Lobde-HrTH in *Episymploce sundaica*. Peaks were labeled according to the b- and y-ion series as calculated in Supplementary Figure S10. For original spectra, see Supplementary Figure S11.

**Figure 7:**
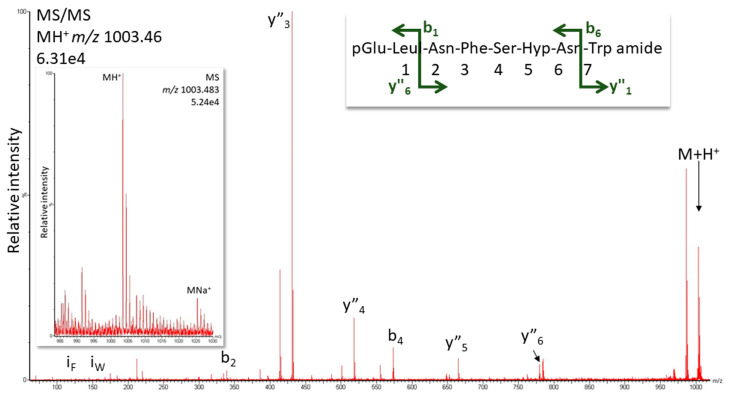
CID analysis for [Hyp^6^]-Tenmo-HrTH in *Ergaula capucina* using the singly charged peptide ion (*m*/*z* 1003.46). Peaks were labeled according to the b- and y-ion series as calculated in Supplementary Figure S12. For original spectrum and validation with the synthetic compound, see Supplementary Figure S13. Inset: Overview spectrum showing the protonated and sodiated peptide peaks.

*K. flavicollis* belongs to the order Blattodea, which is comprised of termites and cockroaches; interestingly, all Blattodea AKHs (from 85 species [54]) have Pro in position 6 and Manto-CC is the octapeptide core structure of the decapeptide Bladi-HrTH (see Table 1) that is prevalent in the cockroach Blaberoidea superfamily. The discovery of Hyp forms for Bladi-HrTH and several related decapeptides [13] is, therefore, not entirely unexpected. Here we supply validation of the [Hyp^6^] decapeptides first reported from cockroach CC extracts recently [13]. Figure 4 shows the CID spectrum of the singly charged ion for [Hyp^6^]-Bladi-HrTH measured in the CC extract of *Xestoblatta cavicola* (see Supplementary Figure S5 for spectrum from *Eublaberus posticus*). Further spectra of peptides related to Bladi-HrTH are presented for [Hyp^6^]-Panni-HrTH found in *Panchlora nivea* (Figure 5), for [Hyp^6^]-Lobde-HrTH from *Episymploce sundaica* (Figure 6), and for [Hyp^6^]-Asiky-HrTH and [Hyp^6^]-Blaat-HrTH from *Asiablatta kyotensis* and *Blaberus atropos*, respectively (Supplementary Figure S9). The sequences of these decapeptides differ only slightly or not at all in the recognition sequence trio (SPG, Table 1). [Hyp^6^]-Tenmo-HrTH (octapeptide) was detected in *E. capucina* and validated [13], but had a comparatively weak spectral response (Figure 7), possibly due to the recognition sequence SPN. Hyp was not detected in the synthetic standards of Manto-CC and Tenmo-HrTH, but at low levels in Bladi- and Blaat-HrTH. Product quality and purity apparently differed among the commercial products.

As we have now made it a habit to screen for the Hyp form of Pro-containing AKHs, we have also found a representative in the grasshopper, *Tetrix subulata* (a Hyp AKH with the recognition sequence TPG). It is known that *T. subulata* CC contain two peptides: Schgr-AKH-II, which has no Pro, and Tetsu-AKH, which does [55]. Evidence for low concentrations of [Hyp^6^]-Tetsu-AKH was obtained in the current study (i.e., candidate peptide measured at expected mass and at earlier retention time than Tetsu-AKH, mass shift of the y″_3_ fragment ion *m*/*z* 358.19 > 374.18), whereas no oxidative modification was found in Schgr-AKH-II (also not for Trp). The CID spectrum did not contain sufficient fragment ions for confident assignment and is not shown, but the features that were measured agreed with those of the synthetic peptide [Hyp^6^]-Tetsu-AKH.

We estimated the relative abundance of the Hyp forms based on the peak area of the extracted ion for the singly charged peptides in the overview MS scan, as well as for a dominant characteristic fragment ion in MS/MS data, and found them to vary between 0.3 and 7.3%. Clearly, in cases of low concentration of the AKH itself, its Hyp form may simply not be measurable, because it falls below the level of detection. For instance, in the CC extract of the American cockroach *Periplaneta americana*, we found some indication for Hyp of the endogenous octapeptides Peram-CAH-I and -II (i.e., a singly charged peptide ion peak measured at the expected mass, an earlier retention time in LC than the “parent” peptide, and the correct y″_3_ fragment ion), but the CID spectra were not of sufficient data content for a confident assignment of the Hyp forms of these two octapeptides. The CC extracts of different species vary greatly with respect to the size of the organ, the concentration of the stored AKHs, and the presence of matrix substances. However, numerous cockroach species are easily commercially available and samples with comparatively high concentrations of AKHs can be prepared so that they are a good source to study the phenomenon of hydroxyprolination further.

#### 2.1.2. Artifact or Natural Product?

In order to confirm that Hyp was not introduced during sample handling, we performed a dedicated experiment using freshly prepared CCs from the cockroach species *B. atropos* and *P. americana*. Tissue preparation, AKH extraction, and MS analysis were carried out on the same day and, in addition, samples were handled under a nitrogen atmosphere whenever possible. Moreover, we processed Bladi- and Blaat-HrTH synthetic standards alongside in the same manner. We detected all four AKHs in the CC extract: Bladi- and Blaat-HrTH and their Hyp forms (for LC and CID data, see Supplementary Figure S14). Thereby, Blaat-HrTH was only present at about a tenth of the Bladi-HrTH concentration as estimated based on ion intensities as explained above. Accordingly, the data for the Hyp form of Bladi-HrTH were much more intensive than those for [Hyp^6^]-Blaat-HrTH, but the latter was clearly present as indicated by marker fragment ions. The peptides were validated using the synthetic Hyp species (Supplementary Figure S15).

The synthetic peptides we processed alongside the CC extracts under nitrogen contained minor amounts of Hyp to begin with (possibly residues from synthesis), which is not a problem for the normally desired purpose of AKH validation, but was disadvantageous here. However, we did not see an increase in the concentration of the Hyp forms during the experimentation, i.e., the extraction and drying procedure did not result in additional hydroxyprolination.

Furthermore, in parallel, we dissected CC from *P. americana* for the same purpose. Again, from freshly prepared CC, the identification of the Hyp form of the AKHs was as ambiguous as the data obtained from earlier samples taken in a more routine procedure as discussed above. Enzyme recognition trios are SPN and TPN for Peram-CAH-I and -II, respectively, and these are possibly not as effective as XPG sequences, as we have noted for Tenmo-HrTH also (recognition sequence SPN). In general, however, the recognition sequences found here fit well with those observed thus far in the literature (Table 2). These results strongly hint at an enzymatic process. Importantly, we did not “generate” Hyp during sample processing of freshly prepared *Periplaneta* CCs, because we would then have seen similar amounts as for *Blaberus* Hyp peptides.

In the current study, we also induced the more natural release of peptides from the CC through the process of membrane depolarization ex vivo, instead of via the physical disruption of the CC cells by sonification, to establish if the Hyp peptides were releasable as confirmation of being true hormones. Thus, *B. atropos* CCs were carefully removed from the insect body to remain intact and incubated in a saline solution containing a high Ca^2+^ and K^+^ concentration [56]; the saline was retained for LC-MS. In such experiments, about 10% of the content of the CC can be released, which is much less peptide material than extracted under our normal CC preparation. The lower content was reflected in the LC-MS data; nevertheless, all four peptides were clearly detected (Supplementary Figure S16), underlining our interpretation that the two Hyp forms of the decapeptides are not artificially introduced but were synthesized and released from CC neurosecretory cells through depolarization of the cell membrane.

#### 2.1.3. Bioactivity

As summarized in Table 3, all the tested [Hyp^6^]-containing peptides were able to elevate the level of total carbohydrates in the hemolymph significantly when 10 pmol was injected into the acceptor cockroach *P. americana* at rest, whereas control injections with water had no significant effect. Injection of a positive control, i.e., a tenth of a CC extract of the acceptor cockroach *P. americana*, or the synthetic version of one of the endogenous peptides of *P. americana* (Peram-CAH-I) of this insect, also increased the circulating carbohydrates significantly. It is clear that [Hyp^6^]-containing peptides demonstrate hypertrehalosemic activity; in the case of Bladi- and Blaat-HrTH, there is a somewhat reduced biological response to the hydroxyprolinated versions. This is, however, not significant. In all of the peptides tested, Blaat-HrTH and its Hyp form consistently stimulate the weakest hypertrehalosemic response in *P. americana* (Table 3 and ref. [13]).

### 2.2. Tryptophan Oxidation

**Figure 8:**
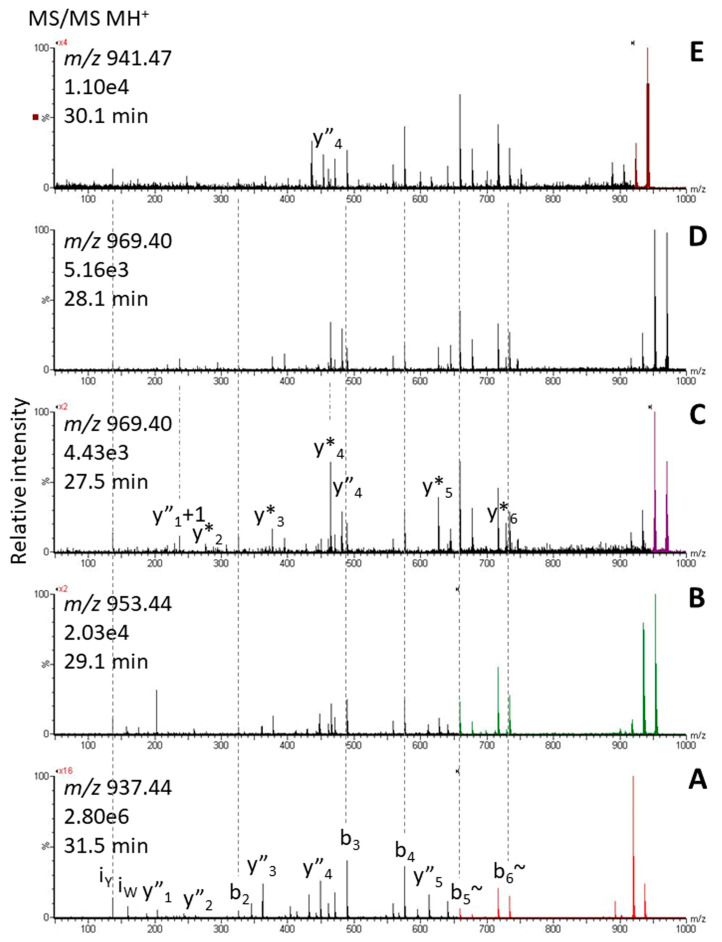
CID spectra of the singly charged ions of a peptide assigned to Nicve-AKH in *N. orbicollis* (**A**) and its singly (**B**) and doubly oxidized (**C**,**D**) as well as the Kyn-form (**E**). For spectra and expected ions, see Supplementary Figures S17 and S18.

An octapeptide with an unusual tyrosine residue at position 4 was previously characterized from the burying beetle *Nicrophorus vespilloides* and code-named Nicve-AKH: pQLTYSTGW amide [57]. When additional species of *Nicrophorus* subsequently became available (as part of an investigation into the biodiversity of AKHs in polyphagan beetles), we observed considerable oxidation of the Trp residue of Nicve-AKH in all four species investigated. The peptide was modified by one and two oxygens as well as the loss of CO forming Kyn (Figure 2); for example, see Figure 8 for results from *N. orbicollis*. We did not check for other oxidation products [36] which may have been there. For the doubly oxidized Trp, we noted products at two different retention times (about 0.5 min apart). They likely resulted from the two possible products dioxindoylalanine and *N*-formylkynurenine (Figure 2), which can be formed via a dioxetane intermediate or a hydroperoxide function [15,35]. Their CID spectra differed only slightly, with the later eluting form (Figure 8D) losing ammonia more easily than the other (Figure 8C). On a side note, two distinct peaks have been observed for oxindoylalanine-containing small peptides by other authors, and the hindrance of keto-enol tautomerism by neighboring amino acid residues was given as a possible reason [34].

The peptides bearing the various Trp modifications for Nicve-AKH were measured from well below 1% up to 7.2%. Of the tested oxidation products, the singly oxidized species was the most abundant form (1.2–7.2%, average 3.7%) while the doubly oxidized species was present at ~0.3% and Kyn at ~0.6%. For the synthetic AKH, no oxidation products were detected in a quick check. Control experiments, which favor artificial oxidation during handling have, however, not been performed.

It is rare that such abundant Trp modifications are reported in samples and can be taken as a hint that endogenous post-translational Trp oxidation is unlikely in this case. It should be said that, ordinarily, no extra precautionary measures are taken during the handling of the CC, such as the addition of reducing agents, and should perhaps be considered, especially before samples are sent (by post) to destinations 10,000 km away. The *Nicrophorous* samples examined in the current study had, in fact, traveled to several laboratories around the world as an exceptional case. Further tests are required with freshly dissected CC material. However, in all other CC extracts re-examined in the current study (and reported on in Section 2.1.1 above), none displayed measurable Trp oxidation products.

## 3. Materials and Methods

### 3.1. Insects and CC Preparation

Adult males of the cockroach *B. atropos* were purchased from a commercial dealer, while adult males of *P. americana* were a gift from the research group of Prof. R. Predel (University of Cologne, Cologne, Germany). Cockroaches were held at 30 ± 2 °C, RH at 60%, 14 h light:10 h dark cycle and fed with a mixture of dog and rabbit food plus water ad libitum. *P. americana* was used as an acceptor insect for the biological assays (see Section 3.2 below). *B. atropos* and *P. americana* were also used in experiments to ascertain the nature/origin of the Hyp-modification of AKHs in cockroaches (see Section 3.3 and Section 3.5 below for CC preparation details).

For AKH peptide structure elucidation and for use in biological assays, CCs were dissected from adult insects of indeterminate age. CC from individual insects (n = 4–6) were dissected with the aid of a stereomicroscope at 20- to 40-fold magnification. The glands from the same species were pooled in a microcentrifuge tube containing 80% methanol, extracted as described [58], and dried in a vacuum centrifuge. All cockroach species were purchased from commercial breeders with exceptions: *P. americana* (see above), *Anaplecta* ssp, and *X. cavicola* were gifted by the research group of Prof. P. Deleporte (University of Rennes, Rennes, France), and *Aptera fusca* specimens were caught on the premises of the University of Cape Town, Cape Town, South Africa. Specimens of the termite *K. flavicollis* (Blattodea, Isoptera, Kalotermitidae) were a gift from Prof. D.P. McMahon (Federal Institute of Materials Research and Testing, BAM, Berlin, Germany). CC extracts from the pygmy grasshopper, *T. subulata*, were prepared in 2014 from specimens that were collected near Osnabrück. Burying beetles of the genus *Nicrophorus* were obtained as a gift from Prof. S. Steiger (Evolutionary Animal Ecology, University of Bayreuth, Bayreuth, Germany). The CCs were prepared from *N. pustulatus*, *N. orbicollis*, *N. quadripunctatus,* and *N. defodicus* in 2017, and the dried crude extracts were subjected to high ambient temperatures during atypical episodes of courier transportation prior to MS investigation in the current study.

### 3.2. Biological Assay

A dried methanolic CC extract from *P. americana* was reconstituted in distilled water and injected in resting *P. americana* males as a positive control. Synthetic peptides were dissolved in 40 µL of 50/50 *v*/*v* methanol and 0.1% formic acid containing 5% acetonitrile and then diluted 1:1000 with distilled water for injection of 10 pmol peptide in a volume of 10 µL into *P. americana* to measure their hypertrehalosemic activity as previously outlined in detail [13] (with the exception that experimentation took place at 26 ± 2 °C).

### 3.3. Release Experiment

Seven CCs from *B. atropos* were dissected free of adhering tissues and placed into an Eppendorf tube with 50 µL of saline consisting of a high concentration of calcium and potassium [56]. The tube was left shaking gently (300 rpm) for 30 min at 25 °C. Thereafter, the saline was carefully removed from the tube, leaving the intact CC behind; an aliquot of the saline was desalted by solid-phase extraction (Zip-tip C18, Millipore, Burlington, MA, USA), dried, and redissolved in 5 µL 50/50 *v*/*v* methanol and 0.1% formic acid containing 5% acetonitrile for LC-MS analyses.

### 3.4. Synthetic Peptides

Besides the *Blaberus* peptides (Bladi- and Blaat-HrTH, and their [Hyp^6^]-modified forms), we ordered synthetic peptides for [Hyp^6^]-Manto-CC because of its high structural similarity to Bladi-HrTH and its relatives, which share the same recognition sequence, and [Hyp^6^]-Tenmo-HrTH for the presence of Asn instead of Gly in the recognition trio (Table 1). The hydroxylated forms of Manto-CC and Tenmo-HrTH were synthesized by Peptide Specialty Laboratory GmbH (Heidelberg, Germany, HPLC-purified), whereas the hydroxylated forms of Bladi-HrTH and Blaat-HrTH came from Pepmic Co., Ltd. (Suzhou, China, 85% purity) as did the octapeptides Nicve-AKH, Tetsu-AKH, and Schgr-AKH-II, and the decapeptide Blaat-HrTH. Peram-CAH-I and Bladi-HrTH originated from Peninsula Laboratories (Belmont, CA, USA, 85% purity). Stock solutions of these peptides were prepared at 1 pmol/µL in 50/50 *v*/*v* methanol and 0.1% formic acid containing 5% acetonitrile.

### 3.5. Precautions against Oxidation

CC were dissected from *B. atropos* and *P. americana* (5 specimens each). Tissue preparation, AKH extraction, and MS analysis were performed on the same day, and, in addition, samples were handled under a nitrogen atmosphere whenever possible to limit oxidation events. Moreover, synthetic Bladi-HrTH and Blaat-HrTH (20 pmol each) were processed alongside the CC material in the same manner.

### 3.6. Structure Elucidation by LC-MS

The dried CC extracts from various insect species were dissolved in 10 µL methanol followed by 10 µL 0.1% formic acid containing 5% acetonitrile. For LC-MS/MS, Synapt G2 Si (Q-TOF with ion mobility) coupled to M-Class nanoUPLC (Waters Corp., Manchester, UK) was employed using C18 µPAC columns (trapping and 50 cm analytical; PharmaFluidics, Ghent, Belgium) with a 30 min gradient (10–60%; solvent system 100% water versus 100% acetonitrile, both containing 0.1% formic acid; 0.4 µL/min flow rate; 0.5–1 µL injection volume). AKH candidates were identified by target-MS (MS/MS on pre-selected *m*/*z* values) using singly and doubly charged ions, as well as by screening with low/high collision energy switching for the gas phase loss of the Trp immonium ion in data-independent runs. Moreover, AKH candidates were obtained by manual interrogation of data-dependent runs and the use of marker fragment ions discovered for Pro-containing AKHs [53]. Sequence ion assignment was used as calculated by the MassLynx spectrometer software V.4.1, which treats pyroglutamate (Pyr) as terminal modification rather than a modified amino acid, thus creating a label shift for ion assignment by one in comparison to the amino acid number. The fragment ion tables for the spectra shown here are available in the Supplement for clarification. Peptide sequences were validated by comparison of the target MS/MS to the performance of the respective synthetic peptides using identical instrument parameters. For validation, both the endogenous and the synthetic samples were spiked with bradykinin 1–7 (Sigma, Darmstadt, Germany, 1 pmol/µL stock solution) for control of the retention time, which eluted about 6 min earlier than the AKHs. It allowed correction of the LC arrival time following heavy unrelated use of the instrumentation, if necessary.

## 4. Conclusions

We detected Hyp and oxidation products of Trp for AKHs from several insect species. Both can potentially be handling artifacts, but the likelihood of this seems to be much higher for Trp. Hyp is not generated at ambient conditions easily, in contrast to oxidized Trp, for which many reports document spontaneous formation. In addition, much is known about prolyl hydroxylases while knowledge about Trp peptide oxygenases is sparse. Moreover, the oxidized Trp products were only observed in samples that had not been sufficiently cooled during prolonged transit periods.

During the discovery of [Hyp^6^]-Panbo-RPCH [11], precautions were taken to avoid any change in the CC extract as a result of environmental conditions. We also performed dedicated experiments to that end with cockroach CC here and are confident that hydroxyprolination of AKHs is indeed occurring inside the insect CC. It is a substochiometric process, the reason for which has yet to be uncovered. The data so far acquired from insect CC show recognition sequences in [Hyp^6^]-AKHs which are well described in the literature, and which have been ascribed as substrates to P4H. Proof that P4H modifies Pro^6^ in AKHs has, however, yet to be obtained.

We have shown in heterospecific biological assays that synthetic [Hyp^6^]-AKHs are biologically active, causing hypertrehalosemia, albeit at a slightly lower efficiency than the unmodified “parent” peptide; the precise function of the modified peptides in the insects remains to be clarified.

We have also observed oxidized Trp and, in this case, advise caution. It is rare to find such abundant Trp modifications in AKHs and, in this case, we assume storage and handling issues to be the reason. This hypothesis has yet to be proven, but in the light of all available evidence, an endogenous post-translational Trp oxidation appears unlikely. In any case, future research will have to clearly differentiate between in vivo and externally induced processes and find out whether or not there is any functional meaning to the oxidation of Trp in insects, possibly in the context of degradation and aging.

## Figures and Tables

**Table 1:** [Hyp^6^]-AKHs as determined in the current and former studies using high-resolution MS. Sorted by insect species and enzyme recognition sequence. MW—molecular weight.

Species	Sequence	MW	Name
*Nezara viridula*	pQLNF S Hyp G W amide	945.434	[Hyp^6^] Panbo-RPCH [11]
*Kalotermes flavicollis*	pQVNF S Hyp G W amide	931.419	[Hyp^6^]-Manto-CC
*Blaberus atropos*	pQVNF S Hyp G WGT amide	1089.4880	[Hyp^6^]-Bladi-HrTH
*Blaberus atropos*	pQLNF S Hyp G WGF amide	1149.5244	[Hyp^6^]-BlaatHRTH
*Eublaberus posticus*	pQVNF S Hyp G WGT amide	1089.4880	[Hyp^6^]-Bladi-HrTH
*Episymploce sundaica*	pQVNF S Hyp G WGT amide	1089.4880	[Hyp^6^]-Bladi-HrTH
*Episymploce sundaica*	pQLNF S Hyp G WGP amide	1099.5087	[Hyp^6^]-Lobde-HrTH
*Asiablatta kyotensis*	pQVNF S Hyp G WGT amide	1089.4880	[Hyp^6^]-Bladi-HrTH
*Asiablatta kyotensis*	pQLNF S Hyp G WGV amide	1101.5244	[Hyp^6^]-Asiky-HrTH
*Panchlora nivea*	pQVNF S Hyp G WGT amide	1089.4880	[Hyp^6^]-Bladi-HrTH
*Panchlora nivea*	pQLNF S Hyp G WGT amide	1103.5037	[Hyp^6^]-Panni-HrTH
*Supella longipalpa*	pQVNF S Hyp G WGT amide	1089.4880	[Hyp^6^]-Bladi-HrTH
*Loboptera decipiens*	pQVNF S Hyp G WGT amide	1089.4880	[Hyp^6^]-Bladi-HrTH
*Loboptera decipiens*	pQLNF S Hyp G WGP amide	1099.5087	[Hyp^6^]-Lobde-HrTH
*Xestoblatta cavicola*	pQVNF S Hyp G WGT amide	1089.4880	[Hyp^6^]-Bladi-HrTH
*Nauphotea cinera*	pQVNF S Hyp G WGT amide	1089.4880	[Hyp^6^]-Bladi-HrTH
*Aptera fusca*	pQVNF S Hyp G WGT amide	1089.4880	[Hyp^6^]-Bladi-HrTH
*Haematopota pluvialis*	pQLTF **T** Hyp G W amide	946.455	[Hyp^6^]-Haepl-AKH [12]
*Ergaula capucina*	pQLNF S Hyp **N** W amide	1002.4560	[Hyp^6^]-Tenmo-HrTH [13]

Bold indicates unusual Thr and Asn residues in X-Hyp-X sequence parts.

**Table 2:** Evidence for endogenous hydroxyprolinated sites in peptides from the literature and this study.

				References
	**X**	Hyp	**G**	[20,22]
	**P**	Hyp	**G**	[20,22,23,25]
	**S**	Hyp	**G**	this study, [25]
	**T**	Hyp	**G**	this study
	**A**	Hyp	**G**	[23]
	**R**	Hyp	**G**	[24]
	**S**	Hyp	**A**	[25]
	**D**	Hyp	**V**	[25]
	**T**	Hyp	**P**	[26,27]
	**T**	Hyp	**N**	[25]
	**T**	Hyp	**N**	this study
	**S**	Hyp	**N**	this study
	**S**	Hyp	**E**	[25]
	**K**	Hyp	**Q**	[26,27]
	**A**	Hyp	**S**	[25]
	**R**	Hyp	**T**	[28]
**T**	Hyp	Hyp	**K**	[26,27]
	**P**	Hyp	**K**	[26,27]
**T**	Hyp	Hyp	**R**	[26,27]
	**P**	Hyp	**R**	[26,27]
	**D**	Hyp	**R**	[28]

The minimum requirement was given as XPG (marked in light grey) [20,22]. The neighboring residues to Hyp are given in bold. Results from this study are marked in darker grey.

**Table 3:** Biological activity of synthetic peptides in an in vivo bioassay with the American cockroach, *P. americana*. Distilled water and the synthetic equivalent of an endogenous *P. americana* HrTH peptide (10 pmol), as well as a crude methanolic CC extract from *P. americana,* were injected as control substances. Data given as Mean ± SD. * A Paired *t*-test was applied to compare data before and after injection in the same individuals. NS, not significant.

Treatment	*n*	[Carbohydrates] T_0_min (µg/µL)	[Carbohydrates] T_90_min (µg/µL)	Difference (µg/µL)	*p* *
Distilled water	20	17.87 ± 2.94	18.44 ± 3.46	0.57 ± 2.13	NS
Bladi-HrTH	5	20.38 ± 3.89	36.46 ± 9.41	16.08 ± 6.29	0.002
[Hyp]-Bladi-HrTH	19	15.96 ± 2.89	31.06 ± 4.86	15.10 ± 5.64	0.0006
Blaat-HrTH	9	15.44 ± 3.62	24.10 ± 6.51	8.66 ± 6.67	0.002
[Hyp]-Blaat-HrTH	18	17.61 ± 2.75	24.07 ± 1.32	6.46 ± 1.44	0.000001
[Hyp]-Manto-CC	5	17.78 ± 1.44	30.16 ± 4.32	12.38 ± 5.17	0.009
[Hyp]-Tenmo-HrTH	5	18.69 ± 0.95	30.37 ± 1.95	11.67 ± 1.80	0.0001
Peram-CAH-I	9	18.95 ± 2.94	35.92 ± 4.39	16.97 ± 3.61	0.0000003
*P. americana* (0.1 gland pair equivalent)	12	15.00 ± 2.02	45.86 ± 7.06	30.86 ± 6.61	0.000001

## Data Availability

All data are given in the Results and Supplementary Materials sections. Spectra are available at www.uni-muenster.de/Forschungsdaten/angebote/datastore/index.html with doi:10.17879/58938592797, accessible since 1 January 2024.

## Supplementary Materials

The following supporting information can be downloaded at: https://www.mdpi.com/article/10.3390/life13122315/s1, Figure S1: Calculation of the expected fragment ions for sequences pQVNFSPGWa and pQVNFSP(Hyp)GWa of a peptide assigned to Manto-CC (Hyp) in K. flavicollis using MassLynx (blocked termini: pQ and amidation). Note that “~” and “*” refer to the water and ammonia neutral losses; Figure S2: CID spectrum of the singly-charged ion of a peptide assigned to Manto-CC (Hyp) in K. flavicollis (top spectrum for Figure 3 main text). Bottom two spectra: Its validation with the synthetic compound (top trace: termite CC extract, bottom trace: synthetic reference compound); Figure S3: Calculation of the expected fragment ions for sequence pQVNFSP(Hyp)GWGTa of a peptide assigned to Bladi-HrTH (Hyp) using MassLynx (blocked termini: pQ and amidation). Note that “~” and “*” refer to the water and ammonia neutral losses; Figure S4: MS/MS analysis for Bladi-HrTH (Hyp) in *X. cavicola* for the singly- and the doubly-charged peptide ions (*m/z* 1090.50, top trace, and *m/z* 545.75, bottom trace; see Figure 4 main text); Figure S5. MS/MS analysis for Bladi-HrTH (top trace) and its Hyp form in *Eublaberus posticus* for the singly-charged peptide ions; Figure S6. Calculation of the expected fragment ions for sequence pQLNFSP(Hyp)GWGTa of a peptide assigned to Panni-HrTH (Hyp) using MassLynx (blocked termini: pQ and amidation). Note that “~” and “*” refer to the water and ammonia neutral losses; Figure S7. MS/MS analysis for the singly- and the doubly-charged peptide ions (*m/z* 1104.51, top trace, and *m/z* 552.76) of a peptide assigned to Panni-HrTH (Hyp) in *P. nivea* (see Figure 5 main text); Figure S8. Calculation of the expected fragment ions for sequence pQLNFSP(Hyp)GWGVa of a peptide assigned to Asiky-HrTH (Hyp) and sequence pQLNFSP(Hyp)GWGFa for Blaat-HrTH (Hyp) using MassLynx (blocked termini: pQ and amidation). Note that “~” and “*” refer to the water and ammonia neutral losses; Figure S9. MS/MS analysis for the singly-charged peptide ions of peptides assigned to Asiky-HrTH (Hyp) in Asiablatta kyotensis (top trace) and Blaat-HrTH (Hyp) in Blaberus atropos (bottom trace); Figure S10. Calculation of the expected fragment ions for sequence pQLNFSP(Hyp)GWGPa of a peptide assigned to Episu-HrTH (Hyp) using MassLynx (blocked termini: pQ and amidation). Note that “~” and “*” refer to the water and ammonia neutral losses; Figure S11. MS/MS analysis for the singly- and the doubly-charged peptide ions (*m/z* 1100.52, top trace, and *m/z* 550.76) of a peptide assigned to Episu-HrTH (Hyp) in Episymploce sundaica (bottom trace, see Figure 6 main text); Figure S12. Calculation of the expected fragment ions for sequence pQLNFSP(Hyp)NWa of a peptide assigned to Tenmo-HrTH (Hyp) using MassLynx (blocked termini: pQ and amidation). Note that “~” and “*” refer to the water and ammonia neutral losses; Figure S13. CID spectrum of the singly-charged ion of a peptide assigned to Tenmo-HrTH (Hyp) in *E. capucina* (upper trace) and its validation with the synthetic compound (bottom spectrum); Figure S14. LC-MS for the analysis of freshly prepared *Blaberus* CC. Extracted y″_5_ ion traces (top box) and CID spectra. From the bottom in each box: Blaat-HrTH and its Hyp form, Bladi-HrTH and its Hyp form. For expected fragment ions, see Figures S3 and S8; Figure S15. CID spectra for the validation of the Hyp forms of Bladi- and Blaat-HrTH from freshly prepared *Blaberus* CC using the synthetic peptides. From the bottom: synthetic Blaat-HrTH (Hyp) and endogenous peptide, synthetic Bladi-HrTH (Hyp) and endogenous peptide. For expected fragment ions, see Figures S3 and S8; Figure S16. CID spectra for the analysis of freshly prepared *Blaberus* CC extracted with saline. From the bottom: Blaat-HrTH and its Hyp form, Bladi-HrTH and its Hyp form. For expected fragment ions, see Figures S3 and S8; Figure S17. CID spectra of the singly-charged ions of a peptide assigned to Nicve-AKH in *N. orbicollis* (A) and its singly- (B) and doubly-oxidized (C, D) as well as the Kyn-form (E) (see Figure 8 main text); Figure S18. Calculation of the expected fragment ions for sequence pQITYSTGNWa of a peptide assigned to Nicve-AKH using MassLynx (blocked termini: pQ and amidation) and its oxidation products (W + O, W + 2O, Kyn). Note that “~” and “*” refer to the water and ammonia neutral losses.

## Abbreviations

MS—mass spectrometry, MS/MS—tandem MS, LC—liquid chromatography, AKH—adipokinetic hormone, CC—corpora cardiaca, CID—collision-induced dissociation.
