# Supplementary material for: Oxidation Products of Tryptophan and Proline in Adipokinetic Hormones—Artifacts or Post-Translational Modifications?
Source: Life (Basel). 2023 Dec 10;13(12):2315. doi: 10.3390/life13122315 (PMC10744910; doi:10.3390/life13122315)
Supplement: Supplementary file 1 [file life-13-02315-s001.zip › life-2698554-supplementary.pdf]

Article

Oxidation products of tryptophan and proline in adipokinetic hormones – artefacts or posttranslational modifications?

Simone König [1]* Heather G. Marco [2#], Gerd Gäde [2#]

[1] IZKF Core Unit Proteomics, Interdisciplinary Center for Clinical Research, University of Münster, Röntgenstr. 21, 48149 Münster, Germany; koenigs@uni-muenster.de (S.K.)

2 Department of Biological Sciences, University of Cape Town, Private Bag, ZA-7700 Rondebosch/Cape Town, South Africa; heather.marco@uct.ac.za (H.G.M.); gerd.gade@uct.ac.za (G.G.)

\# Shared first authors

* Correspondence: koenigs@uni-muenster.de

b	211.108	325.151	472.220	559.252	656.304	713.326	-
i	72.081	87.056	120.081	60.045	70.066	30.034	159.092
b~	193.098	307.141	454.209	541.241	638.294	695.315	-
b*	194.082	308.125	455.193	542.225	639.278	696.299	-
	1	2	3	4	5	6	7
	Val	Asn	Phe	Ser	Pro	Gly	Trp
	7	6	5	4	3	2	1
y"	-	706.331	592.288	445.220	358.188	261.135	204.114
y~	-	688.321	574.278	427.209	340.177	243.125	186.103
y*	-	689.305	575.262	428.193	341.161	244.109	187.087

b	211.108	325.151	472.220	559.252	672.299	729.321	-
i	72.081	87.056	120.081	60.045	86.061	30.034	159.092
b~	193.098	307.141	454.209	541.241	654.289	711.310	-
b*	194.082	308.125	455.193	542.225	655.273	712.294	-
	1	2	3	4	5	6	7
	Val	Asn	Phe	Ser	Hyp	Gly	Trp
	7	6	5	4	3	2	1
y"	-	722.326	608.283	461.215	374.183	261.135	204.114
y~	-	704.316	590.273	443.204	356.172	243.125	186.103
y*	-	705.300	591.257	444.188	357.156	244.109	187.087

Figure S1. Calculation of the expected fragment ions for sequences pQVNFSPGWa and pQVNFSP(Hyp)GWa of a peptide assigned to Manto-CC (Hyp) in *K. flavicollis* using MassLynx (blocked termini: pQ and amidation). Note that "~" and "*" refer to the water and ammonia neutral losses.

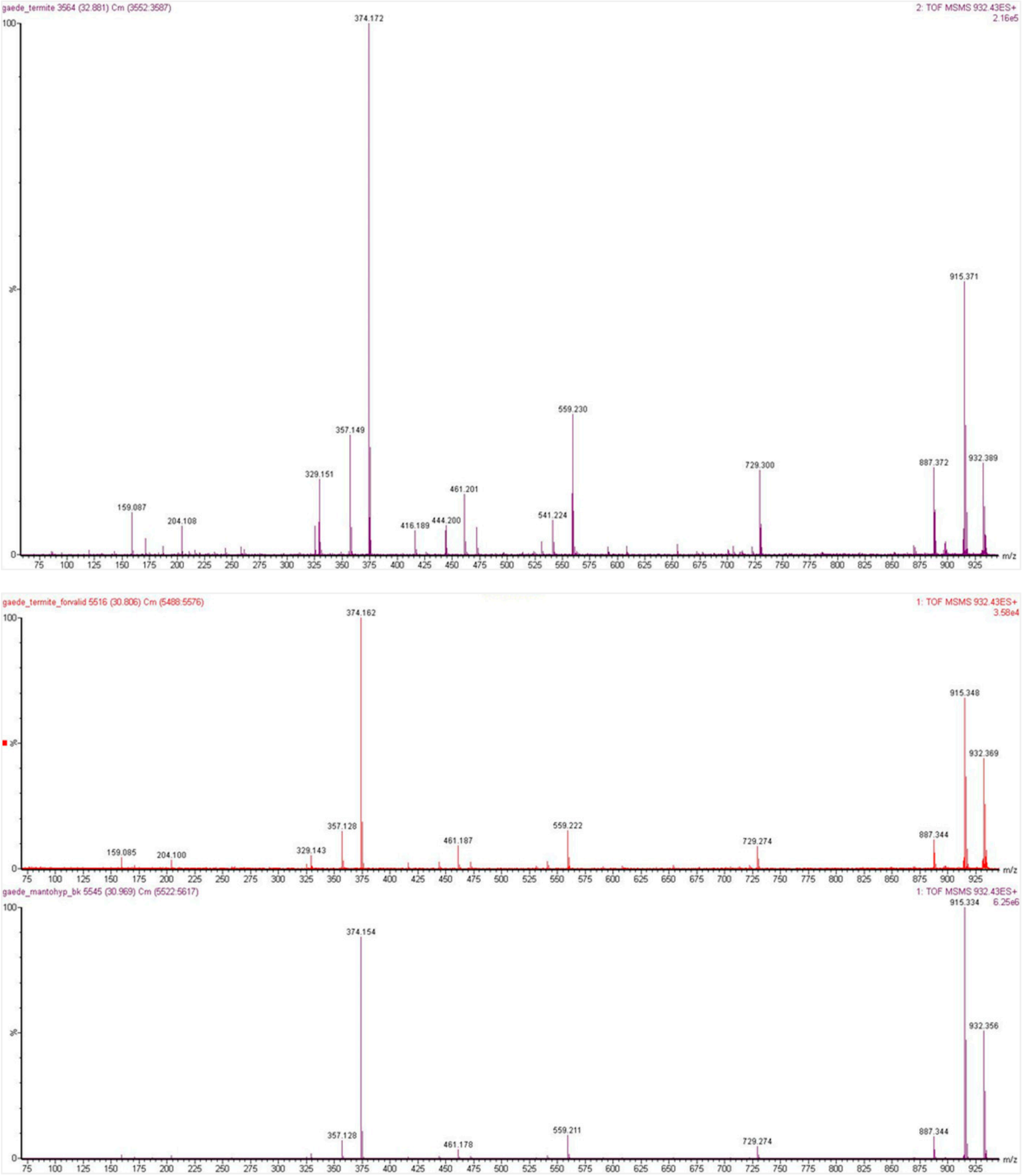

Figure S2. CID spectrum of the singly-charged ion of a peptide assigned to Manto-CC (Hyp) in *K. flavicollis* (top spectrum for Fig. 3 main text). Bottom two spectra: Its validation with the synthetic compound (top trace: termite CC extract, bottom trace: synthetic reference compound).

b	211.108	325.151	472.220	559.252	672.299	729.321	915.400	972.422	-
i	72.081	87.056	120.081	60.045	86.061	30.034	159.092	30.034	74.061
b~	193.098	307.141	454.209	541.241	654.289	711.310	897.390	954.411	-
b*	194.082	308.125	455.193	542.225	655.273	712.294	898.374	955.395	-
	1	2	3	4	5	6	7	8	9
	Val	Asn	Phe	Ser	Hyp	Gly	Trp	Gly	Thr
	9	8	7	6	5	4	3	2	1
y"	-	880.395	766.352	619.284	532.252	419.204	362.183	176.104	119.082
y~	-	862.385	748.342	601.273	514.241	401.194	344.172	158.093	101.071
y*	-	863.369	749.326	602.257	515.225	402.178	345.156	159.077	102.055

Figure S3. Calculation of the expected fragment ions for sequence pQVNFSP(Hyp)GWGTa of a peptide assigned to Bladi-HrTH (Hyp) using MassLynx (blocked termini: pQ and amidation). Note that "~" and "*" refer to the water and ammonia neutral losses.

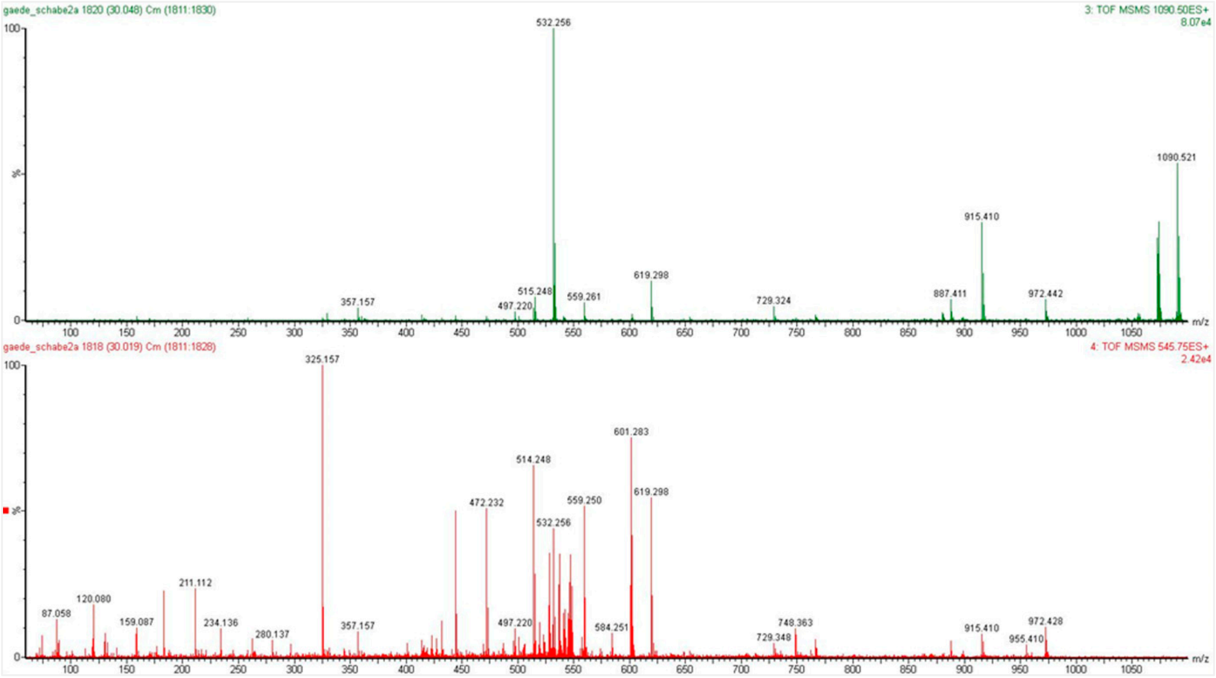

Figure S4. MS/MS analysis for Bladi-HrTH (Hyp) in *X. cavicola* for the singly- and the doubly-charged peptide ions (*m/z* 1090.50, top trace, and *m/z* 545.75, bottom trace; see Fig. 4 main text).

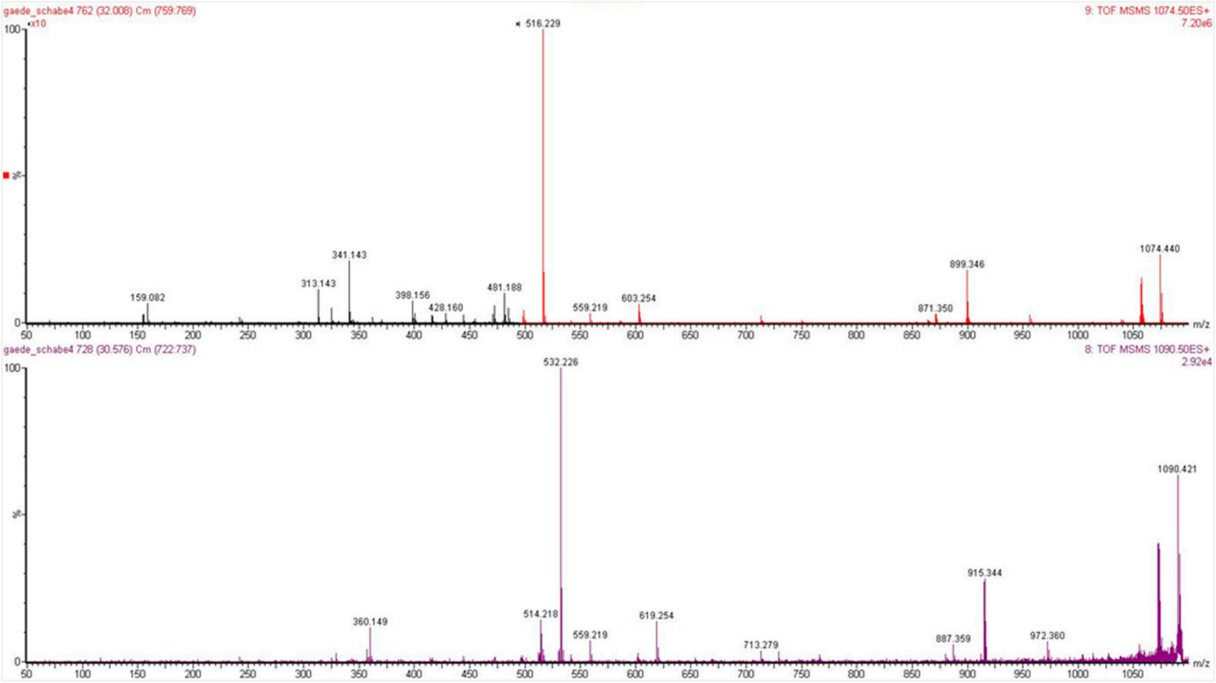

Figure S5. MS/MS analysis for Bladi-HrTH (top trace) and its Hyp form in *Eublaberus posticus* for the singly-charged peptide ions.

b	225.124	339.167	486.235	573.267	686.315	743.336	929.416	986.437	-
i	86.097	87.056	120.081	60.045	86.061	30.034	159.092	30.034	74.061
b~	207.113	321.156	468.225	555.257	668.305	725.326	911.405	968.427	-
b*	208.097	322.140	469.209	556.241	669.289	726.310	912.389	969.411	-
	1	2	3	4	5	6	7	8	9
	**Leu**	**Asn**	**Phe**	**Ser**	**Hyp**	**Gly**	**Trp**	**Gly**	**Thr**
	9	8	7	6	5	4	3	2	1
y"	-	880.395	766.352	619.284	532.252	419.204	362.183	176.104	119.082
y~	-	862.385	748.342	601.273	514.241	401.194	344.172	158.093	101.071
y*	-	863.369	749.326	602.257	515.225	402.178	345.156	159.077	102.055

Figure S6. Calculation of the expected fragment ions for sequence pQLNFSP(Hyp)GWGTa of a peptide assigned to Panni-HrTH (Hyp) using MassLynx (blocked termini: pQ and amidation). Note that "~" and "*" refer to the water and ammonia neutral losses.

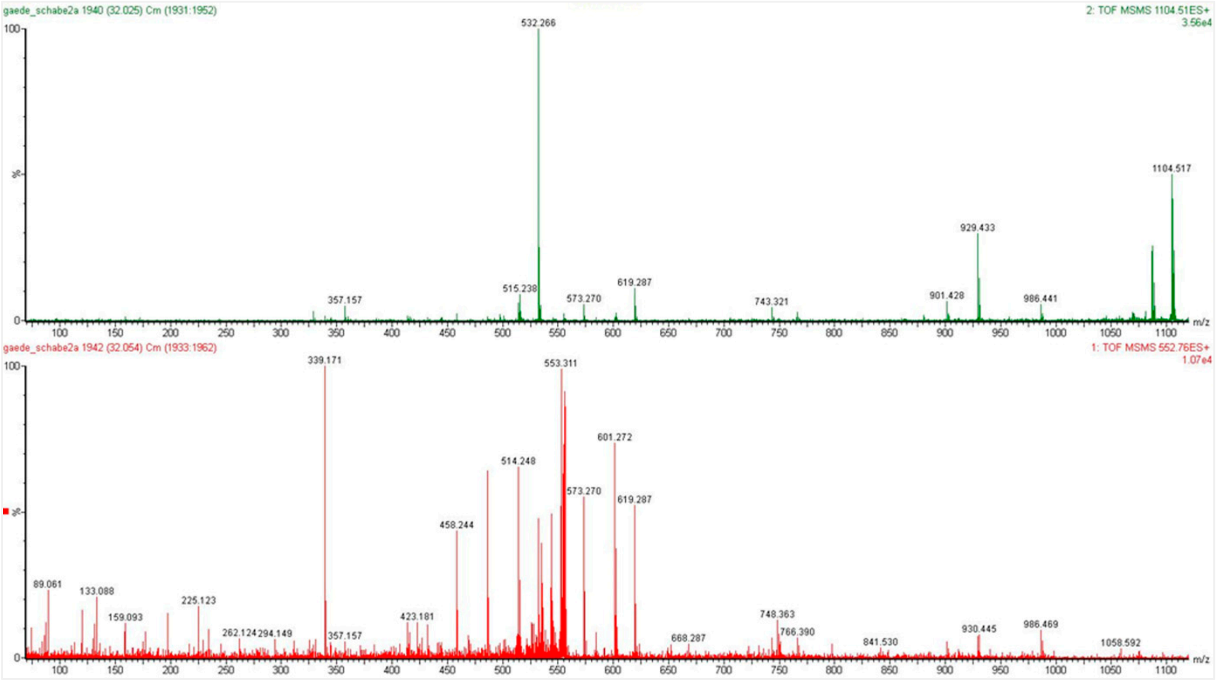

Figure S7. MS/MS analysis for the singly- and the doubly-charged peptide ions (*m/z* 1104.51, top trace, and *m/z* 552.76) of a peptide assigned to Panni-HrTH (Hyp) in *P. nivea* (see Fig. 5 main text).

b	225.124	339.167	486.235	573.267	686.315	743.336	929.416	986.437	-
i	86.097	87.056	120.081	60.045	86.061	30.034	159.092	30.034	72.081
b~	207.113	321.156	468.225	555.257	668.305	725.326	911.405	968.427	-
b*	208.097	322.140	469.209	556.241	669.289	726.310	912.389	969.411	-
	1	2	3	4	5	6	7	8	9
	**Leu**	**Asn**	**Phe**	**Ser**	**Hyp**	**Gly**	**Trp**	**Gly**	**Val**
	9	8	7	6	5	4	3	2	1
y"	-	878.416	764.373	617.305	530.273	417.225	360.204	174.124	117.103
y~	-	860.406	746.363	599.294	512.262	399.214	342.193	156.114	99.092
y*	-	861.390	747.347	600.278	513.246	400.198	343.177	157.098	100.076

b	225.124	339.167	486.235	573.267	686.315	743.336	929.416	986.437	-
i	86.097	87.056	120.081	60.045	86.061	30.034	159.092	30.034	120.081
b~	207.113	321.156	468.225	555.257	668.305	725.326	911.405	968.427	-
b*	208.097	322.140	469.209	556.241	669.289	726.310	912.389	969.411	-
	1	2	3	4	5	6	7	8	9
	**Leu**	**Asn**	**Phe**	**Ser**	**Hyp**	**Gly**	**Trp**	**Gly**	**Phe**
	9	8	7	6	5	4	3	2	1
y"	-	926.416	812.373	665.305	578.273	465.225	408.204	222.124	165.103
y~	-	908.406	794.363	647.294	560.262	447.214	390.193	204.114	147.092
y*	-	909.390	795.347	648.278	561.246	448.198	391.177	205.098	148.076

Figure S8. Calculation of the expected fragment ions for sequence pQLNFSP(Hyp)GWGVa of a peptide assigned to Asiky-HrTH (Hyp) and sequence pQLNFSP(Hyp)GWGFa for Blaat-HrTH (Hyp) using MassLynx (blocked termini: pQ and amidation). Note that "~" and "*" refer to the water and ammonia neutral losses.

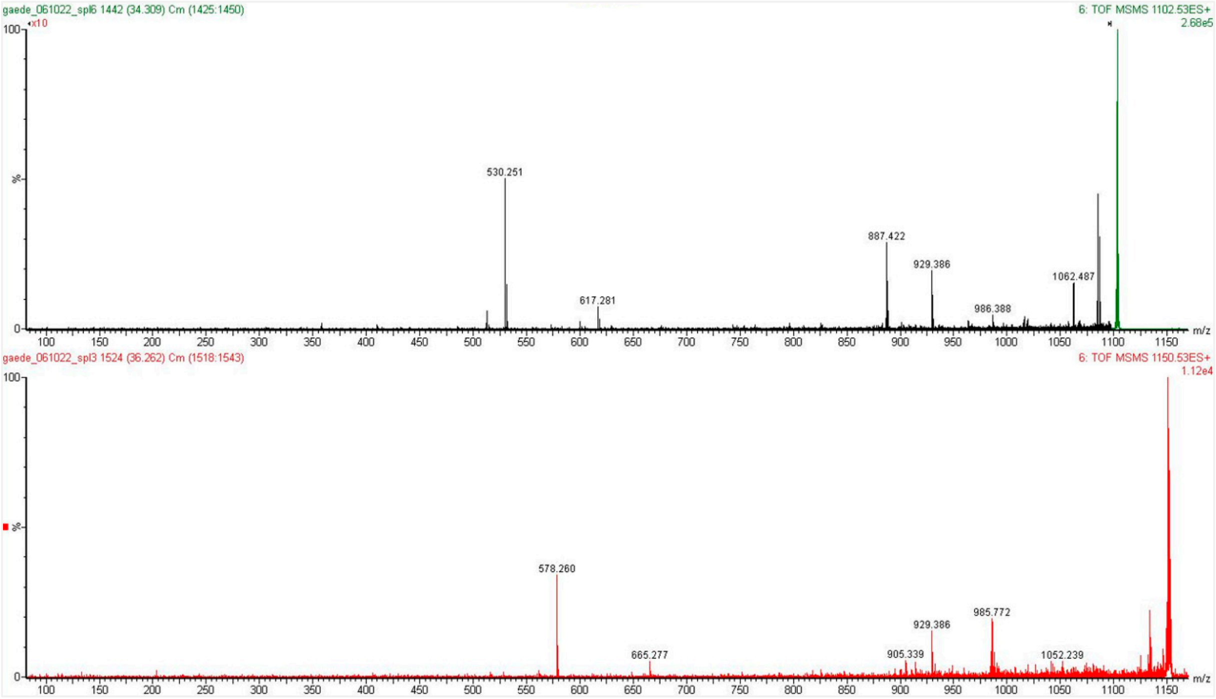

Figure S9. MS/MS analysis for the singly-charged peptide ions of peptides assigned to Asiky-HrTH (Hyp) in *Asiablatta kyotensis* (top trace) and Blaat-HrTH (Hyp) in *Blaberus atropos* (bottom trace).

**b**	225.124	339.167	486.235	573.267	686.315	743.336	929.416	986.437	-
**i**	86.097	87.056	120.081	60.045	86.061	30.034	159.092	30.034	70.066
**b~**	207.113	321.156	468.225	555.257	668.305	725.326	911.405	968.427	-
**b***	208.097	322.140	469.209	556.241	669.289	726.310	912.389	969.411	-
	1	2	3	4	5	6	7	8	9
	**Leu**	**Asn**	**Phe**	**Ser**	**Hyp**	**Gly**	**Trp**	**Gly**	**Pro**
	9	8	7	6	5	4	3	2	1
**y''**	-	876.400	762.357	615.289	528.257	415.209	358.188	172.109	115.087
**y~**	-	858.390	744.347	597.279	510.246	397.199	340.177	154.098	97.077
**y***	-	859.374	745.331	598.263	511.230	398.183	341.161	155.082	98.061

Figure S10. Calculation of the expected fragment ions for sequence pQLNFSP(Hyp)GWGPa of a peptide assigned to Episu-HrTH (Hyp) using MassLynx (blocked termini: pQ and amidation). Note that "~" and "*" refer to the water and ammonia neutral losses.

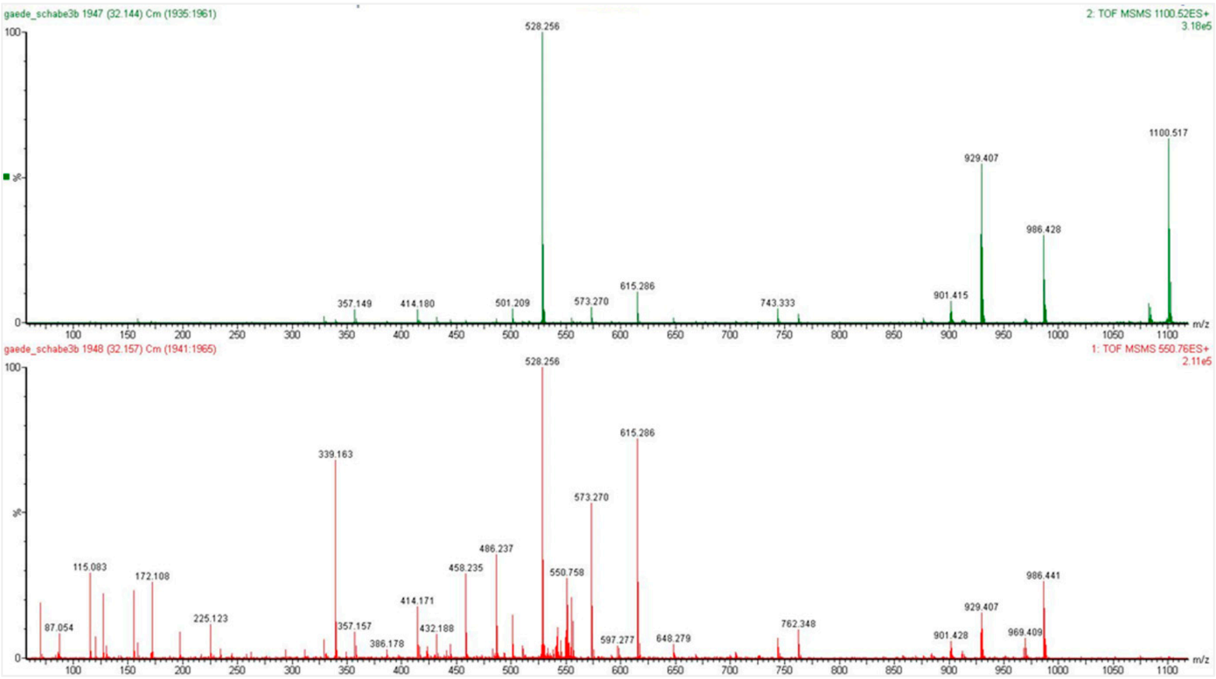

Figure S11. MS/MS analysis for the singly- and the doubly-charged peptide ions (*m/z* 1100.52, top trace, and *m/z* 550.76) of a peptide assigned to Episu-HrTH (Hyp) in *Episymploce sundaica* (bottom trace, see Fig. 6 main text).

b	225.124	339.167	486.235	573.267	686.315	800.358	-
i	86.097	87.056	120.081	60.045	86.061	87.056	159.092
b~	207.113	321.156	468.225	555.257	668.305	782.347	-
b*	208.097	322.140	469.209	556.241	669.289	783.331	-
	1	2	3	4	5	6	7
	**Leu**	**Asn**	**Phe**	**Ser**	**Hyp**	**Asn**	**Trp**
	7	6	5	4	3	2	1
y"	-	779.348	665.305	518.236	431.204	318.157	204.114
y~	-	761.337	647.294	500.226	413.194	300.146	186.103
y*	-	762.321	648.278	501.210	414.178	301.130	187.087

Figure S12. Calculation of the expected fragment ions for sequence pQLNFSP(Hyp)NWa of a peptide assigned to Tenmo-HrTH (Hyp) using MassLynx (blocked termini: pQ and amidation). Note that "~" and "*" refer to the water and ammonia neutral losses.

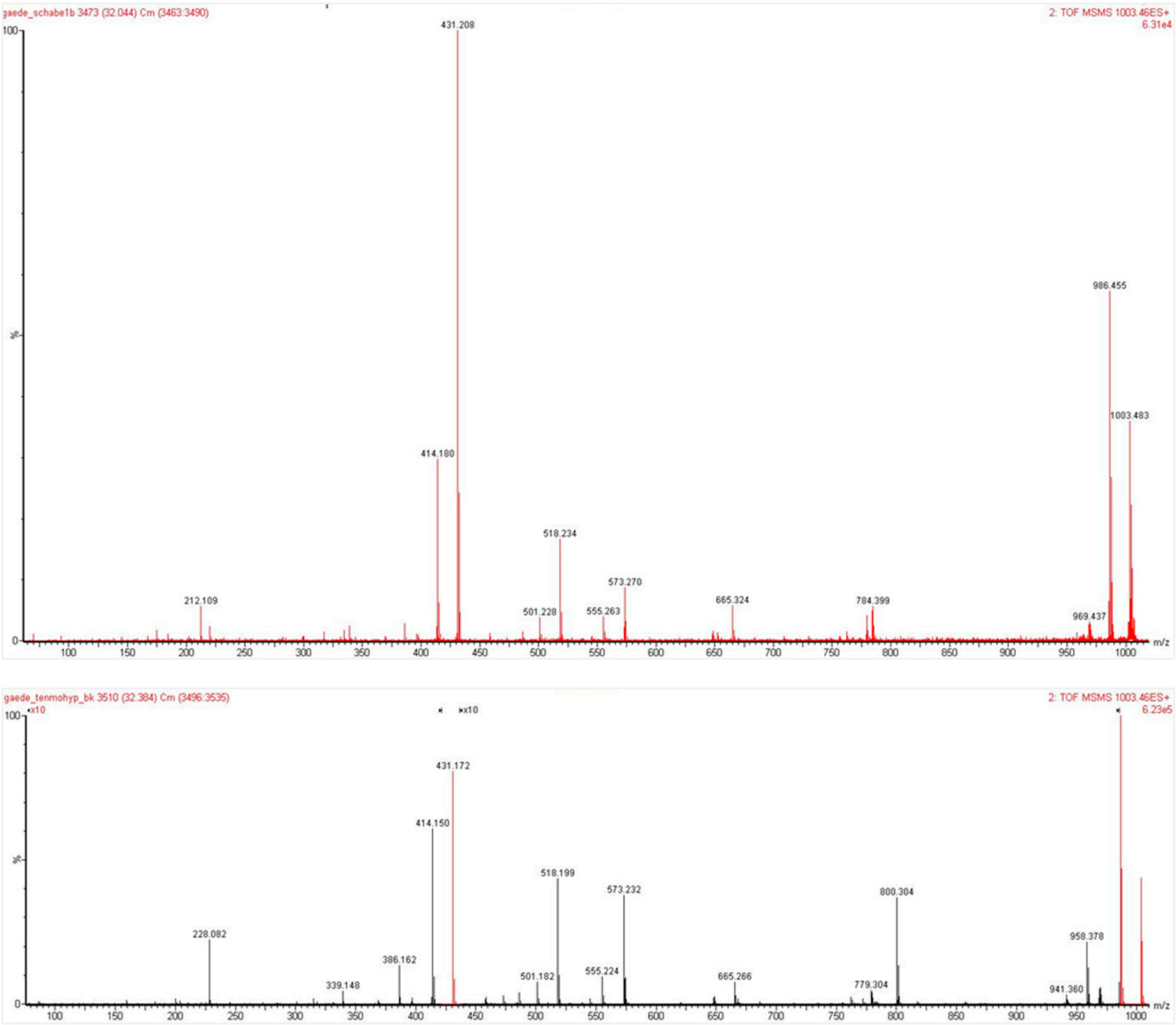

Figure S13. CID spectrum of the singly-charged ion of a peptide assigned to Tenmo-HrTH (Hyp) in *E. capucina* (upper trace) and its validation with the synthetic compound (bottom spectrum).

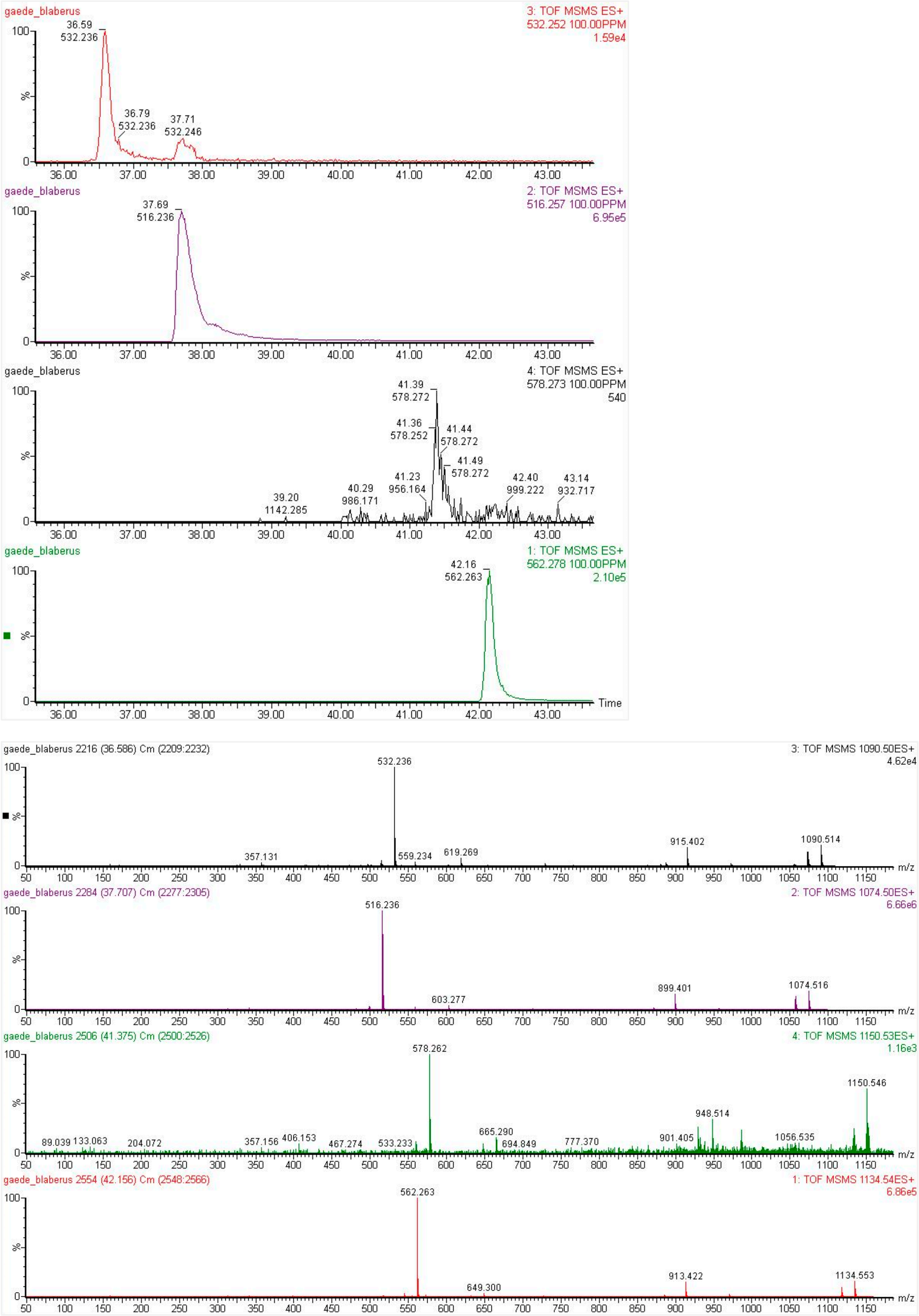

Figure S14. LC-MS for the analysis of freshly prepared *Blaberus* CC. Extracted y”5 ion traces (top box) and CID spectra. From the bottom in each box: Blaat-HrTH and its Hyp form, Bladi-HrTH and its Hyp form. For expected fragment ions, see Figures S3, S8.

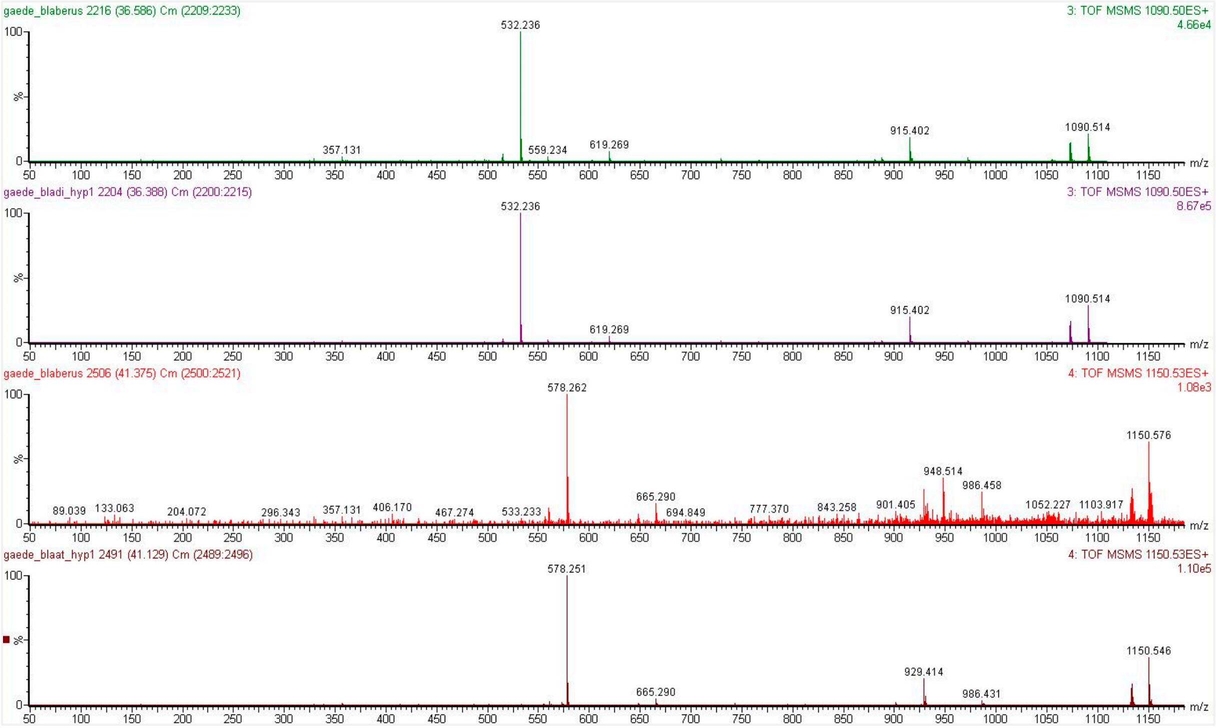

Figure S15. CID spectra for the validation of the Hyp forms of Bladi- and Blaat-HrTH from freshly prepared *Blaberus* CC using the synthetic peptides. From the bottom: synthetic Blaat-HrTH (Hyp) and endogenous peptide, synthetic Bladi-HrTH (Hyp) and endogenous peptide. For expected fragment ions, see Figures S3, S8.

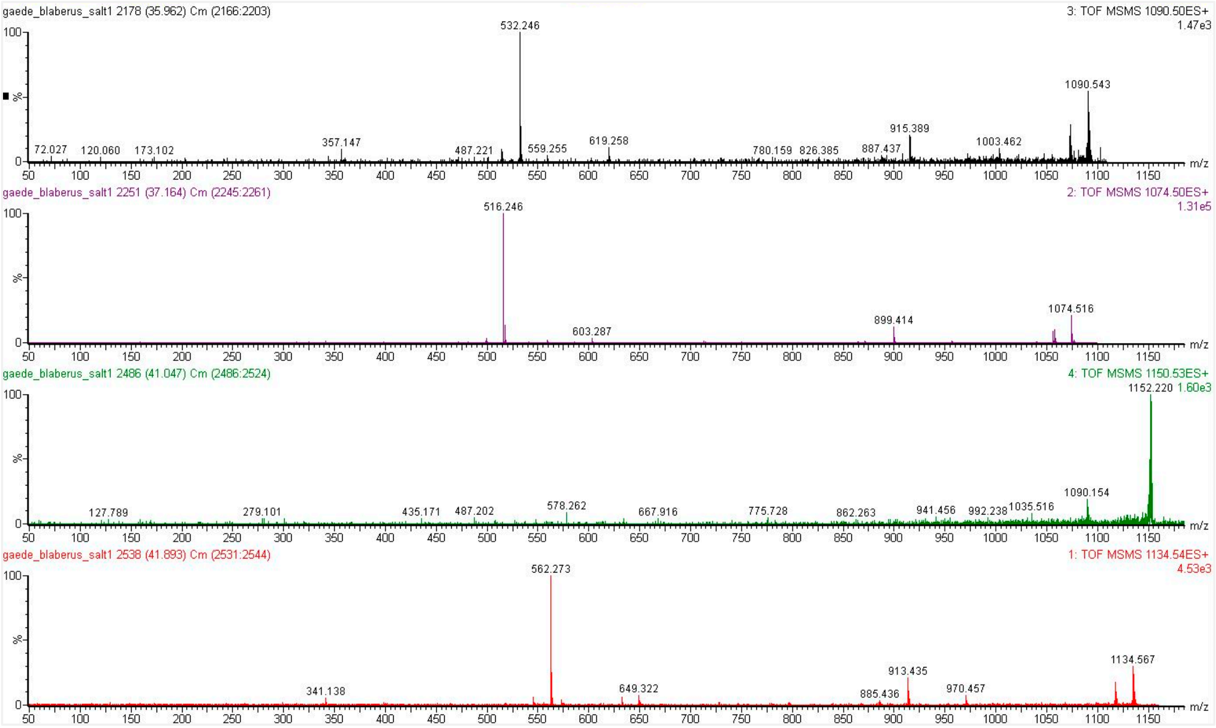

Figure S16. CID spectra for the analysis of freshly prepared *Blaberus* CC extracted with saline. From the bottom: Blaat-HrTH and its Hyp form, Bladi-HrTH and its Hyp form. For expected fragment ions, see Figures S3, S8.

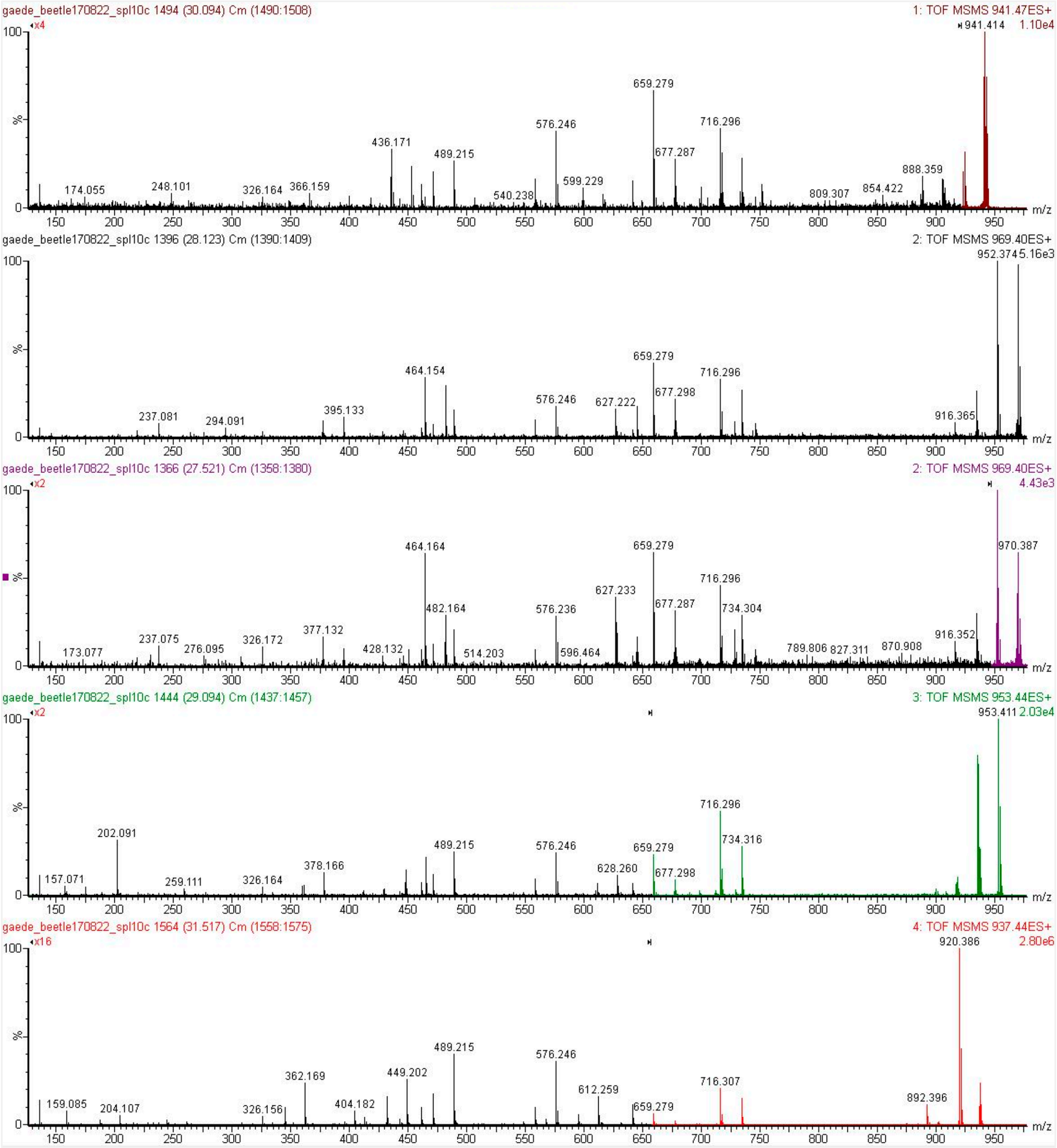

Figure S17. CID spectra of the singly-charged ions of a peptide assigned to Nicve-AKH in *N. orbicollis* (A) and its singly- (B) and doubly-oxidized (C, D) as well as the Kyn-form (E) (see Fig. 8 main text).

b	225.124	326.172	489.235	576.267	677.315	734.336	-
i	86.097	74.061	136.076	60.045	74.061	30.034	159.092
b~	207.113	308.161	471.224	558.256	659.304	716.326	-
b*	208.097	309.145	472.208	559.240	660.288	717.310	-
	1	2	3	4	5	6	7
	Ile	Thr	Tyr	Ser	Thr	Gly	Trp
	7	6	5	4	3	2	1
y"	-	713.326	612.278	449.215	362.183	261.135	204.114
y~	-	695.315	594.268	431.204	344.172	243.125	186.103
y*	-	696.299	595.252	432.188	345.156	244.109	187.087

b	225.124	326.172	489.235	576.267	677.315	734.336	-
i	86.097	74.061	136.076	60.045	74.061	30.034	175.087
b~	207.113	308.161	471.224	558.256	659.304	716.326	-
b*	208.097	309.145	472.208	559.240	660.288	717.310	-
	1	2	3	4	5	6	7
	Ile	Thr	Tyr	Ser	Thr	Gly	Wox
	7	6	5	4	3	2	1
y"	-	729.321	628.273	465.210	378.178	277.130	220.109
y~	-	711.310	610.262	447.199	360.167	259.119	202.098
y*	-	712.294	611.246	448.183	361.151	260.103	203.082

b	225.124	326.172	489.235	576.267	677.315	734.336	-
i	86.097	74.061	136.076	60.045	74.061	30.034	191.082
b~	207.113	308.161	471.224	558.256	659.304	716.326	-
b*	208.097	309.145	472.208	559.240	660.288	717.310	-
	1	2	3	4	5	6	7
	Ile	Thr	Tyr	Ser	Thr	Gly	W2ox
	7	6	5	4	3	2	1
y"	-	745.316	644.268	481.205	394.173	293.125	236.103
y~	-	727.305	626.257	463.194	376.162	275.114	218.093
y*	-	728.289	627.241	464.178	377.146	276.098	219.077

b	225.124	326.172	489.235	576.267	677.315	734.336	-
i	86.097	74.061	136.076	60.045	74.061	30.034	163.124
b~	207.113	308.161	471.224	558.256	659.304	716.326	-
b*	208.097	309.145	472.208	559.240	660.288	717.310	-
	1	2	3	4	5	6	7
	Ile	Thr	Tyr	Ser	Thr	Gly	Kyn
	7	6	5	4	3	2	1
y"	-	717.357	616.309	453.246	366.214	265.166	208.145
y~	-	699.347	598.299	435.236	348.204	247.156	190.134
y*	-	700.331	599.283	436.220	349.188	248.140	191.118

Figure S18. Calculation of the expected fragment ions for sequence pQITYSTGNWa of a peptide assigned to Nicve-AKH using MassLynx (blocked termini: pQ and amidation) and its oxidation products (W + O, W + 2O, Kyn). Note that "~" and "*" refer to the water and ammonia neutral losses.
